# Efficacy of mesenchymal stem cells cultured low oxygen tension ameliorates apoptotic inhibitors, viability, and differentiation of ovarian tissue: A study in a rat model with ovarian failure

**DOI:** 10.12688/f1000research.124919.3

**Published:** 2024-04-16

**Authors:** Erma Safitri, Hery Purnobasuki, Muhammad Thohawi Elziyad Purnama, Shekhar Chhetri

**Affiliations:** 1 Division of Veterinary Reproduction, Department of Veterinary Science, Faculty of Veterinary Medicine, Universitas Airlangga, Surabaya, East Java, 60115, Indonesia; 2 Department of Biology, Faculty of Science and Technology, Universitas Airlangga, Surabaya, East Java, 60115, Indonesia; 3 Division of Veterinary Anatomy, Department of Veterinary Science, Faculty of Veterinary Medicine, Universitas Airlangga, Surabaya, East Java, 60115, Indonesia; 4 Department of Animal Science, College of Natural Resources, Royal University of Bhutan, Lobesa, Punakha, 13001, Bhutan

**Keywords:** malnutrition, ovarian failure, stem cells, good health and well being

## Abstract

**Background:**

In cases of ovarian failure due to malnutrition, stem cell therapy is ineffective due to the lack of viability and differentiation of transplanted cells, resulting in low adaptation and survival rates. This study aimed to investigate whether stem cells cultured under low oxygen (O _2_) tension improve the adaptability and viability of cells in rats with ovarian failure.

**Methods:**

After four days of culturing mesenchymal stem cells (MSCs) in 21% oxygen (normoxia) as the T2 group and 1% oxygen (low O _2_ or hypoxia) as the T1 group, 200 million bone marrow-derived MSCs per rat were transplanted into female rats with ovarian failure (15 rats per treatment group). A total of 15 fertile and 15 infertile rats were categorized as the C+ and C− groups, respectively.

**Results:**

The slight increase gradually in cells expressing HSP70, VEGF-1, and GDF-9 was reported in C+, T2, T1, and C− groups, respectively. Caspase-3 expression was reported to decrease gradually in C+, T2, T1, and C− groups. Meanwhile, Graafian follicle count was reported to increase gradually in C+, T2, T1, and C− groups, respectively.

**Conclusions:**

Based on those parameters, MSCs cultured under low O _2_ tension were more effective than those cultured under normoxic conditions as a treatment for female rats with ovarian failure.

## Introduction

Stem cell transplantation has been explored as a therapy for various diseases such as stroke, ^1^ Alzheimer’s disease, ^2^ diabetes mellitus, ^3^ Parkinson’s, ^4^ myocardial infarction, ^5^ HIV, ^6^ testicular failure, ^7^^,^^8^ and other degenerative diseases such as ovarian failure. ^9^ As an important organ in female reproduction, the ovary produces ovum and female hormones. ^10^ MSC transplantation derived from rabbit bone marrow, ^11^ rat bone marrow, ^7^^,^^9^ rat adipose tissue, ^12^ or umbilical cord blood ^13^ has shown promising results for tissue repair via the proliferation and development of endogenous stem cells into germ cells. MSCs have the ability to differentiate into a variety of cell types, including adipocytes, myocytes, chondrocytes, and osteoblasts. Nevertheless, MSCs can now be applied to develop into germ cell lineage or MSCs that support germ cell lineage regeneration. MSCs exhibit the phenomenon of transdifferentiation, demonstrating their multipotent qualities. MSCs have the ability to develop into a variety of cell types, including ectoderm from mesoderm, neuronal cells, endoderm, and germline cells such as Graafian and growing follicles. ^7^^,^^9^^,^^14^

However, the lack of viability and differentiation of implanted stem cells results in poor adaptivity and a low survival rate, indicating that the therapy is ineffective. The low survivability and function of MSCs might be related to a higher incidence of apoptosis during culture and after transplantation. This finding indicates that the milieu of damaged tissues is not conducive to stem cell adaptation survival; hence, stem cell transplantation is not a feasible option. Recently, researchers cultivating stem cells under low oxygen tension have observed cell stress, although one study has found that the stress situation activates HSP27 as a function of anti-apoptosis via caspase-9 suppression. ^15^

The success of stem cell transplantations is limited by their weak adaptivity and differentiation. The low efficacy of MSCs transplantation might be due to apoptosis occurring during cell growth. ^15^^,^^16^ Consequently, high doses of stem cells via regular boosters are necessary for effective therapy, which increases the therapy cost. ^17^ *In vitro* stem cell cultivation must be adapted to a niche environment, such as the microenvironment of bone marrow, to avoid apoptosis. Low O _2_ tension is an example of a niche habitat for stem cells in bone marrow. ^18^ By reducing apoptosis, viability, and differentiation of stem cells during *in vitro* culture, low O _2_ tension enhances ovarian function in the initiation of ovulation through folliculogenesis and oogenesis. The expression of HSP70 ^19^ and caspase-3 ^20^ in ovarian tissue induces apoptosis, whereas that of VEGF-1 and GDF-9 promotes viability and differentiation. ^21^

This study aimed to determine the role of HSP70 and caspase-3 as apoptotic indicators, as well as VEGF-1 and GDF-9 as viability and differentiation markers, respectively, in MSCs cultivated under low O _2_ tension for malnutrition-induced ovarian failure in female rats. During the in vitro investigation, we observed that the MSC culture under low O _2_ tension enhanced their biological properties. These findings imply that low oxygen tension culture may be a helpful strategy for boosting the effectiveness of MSC-based cell treatments.

## Methods

### Ethics statement

This research, including experiments with animals, was approved by the ethical clearance committee of the Animal Care and Use Committee of the Veterinary Medicine Faculty, Universitas Airlangga with number 239-KE. Experiments were performed in adherence with the guideline manual by the ethical committee. All efforts were undertaken to ameliorate animal suffering.

### Stem cell extraction and culture

Bone marrow stem cells were extracted by aspirating the iliac crest ^7^^,^^9^ of three-month-old male rats. ^22^ The aspirate was placed in heparinized tubes (Z181099, Sigma Aldrich ^®^, Burlington, Massachusetts, USA), which was then transported to the lab and kept at 4°C. ^7^^,^^9^^,^^23^ The aspirate was transferred to 15-mL sterile tubes (SIAL0790-500EA, Sigma Centrifuge tubes, Sigma Aldrich, Burlington, Massachusetts, USA), cleaned two times with 5 mL of sterile phosphate-buffered saline (PBS; MFCD00131855, Sigma Aldrich, Burlington, Massachusetts, USA), and then filled to a final volume of 10 mL. The same amount of Ficoll (F9378, Sigma Aldrich, Burlington, MA, USA) was added after diluting the sample in a separate 15-mL tube. Centrifugation (Sorvall MX Series Floor Model Micro-Ultracentrifuge, Thermo Fisher, Grand Island, NY, USA) was performed at 1,600 rpm for 15 min at room temperature. The mononucleated cells were collected in a 15-mL tube after centrifugation in a form of a “buffy coat” that had accumulated on the Ficoll–PBS surface. In PBS, the cells were resuspended in a 15-mL total volume. The tube (CLS6791 Sigma, Corning LSE Benchtop Shaking Incubator with Platform, Sigma Aldrich, Burlington, MA, USA) was inverted gently and shaken five times to homogenize the suspension.

For another 10 min, the suspension was centrifuged. The cell pellet was resuspended in 6 mL of an alpha-modified essential medium after the supernatant and floating cells were removed from the experiment (α-MEM; M0894; Sigma Aldrich, Burlington, MA, USA). Mononucleated cells were plated in a 10 cm ^2^ plate (Falcon™, Thermo Fisher Scientific, Pittsburgh, PA, USA) with approximately 2×10 ^7^ cells and incubated at 37°C in a humidified atmosphere containing 5% CO _2_ (BioSpherix, Canada/USA) for 24 h to allow cell adherence. Medium and non-adherent cells were removed after 24 h. The plate was returned to the incubator after the adhering cells had been rinsed two times with 5 mL of PBS and 10 mL of fresh α-MEM media. An inverted microscope was used to monitor the culture every day. The medium was replaced every four days, followed by washing with 10 mL of PBS before adding 10 mL of fresh α-MEM media. The culture was maintained until a convergence of about 75%–80% confluence was achieved. The cells were moved to various dishes after confluence to grow subcultures. ^7^^,^^15^ The cells were separated into two low-O _2_-tension treatments of 1% after three rounds in a hypoxic chamber (BioSpherix, Canada/USA) inside a 5% CO _2_ incubator. A separate treatment was the use of 21% O _2_ concentration (normoxic chamber) over four days. MSCs were observed and characterized by using a microscope before being transferred into an ovary. ^7^

### MSCs characterization

MSCs characterization between low O _2_ and normoxic culture conditions was performed before being transferred into the ovary based on morphological analysis of cells using an inverted microscope (Phase Contrast, MXD-400, Nanjing BW Optics & Instrument Co., Ltd). HSP70 and caspase-3 were identified via real-time polymerase chain reaction (PCR; MxPro software, PCR, [Cat No. 401513]; Brilliant II QPCR Low ROX Master Mix [Cat No. 600806], Agilent 2100 Bioanalyzer system [Cat No. G2939AA], Macherey-Nagel GmbH & Co [Düren, Germany]). Apoptosis was detected using immunofluorescence (Apoptag Fluorescein In Situ Apoptosis Detection Kit, S7110, Sigma Aldrich, Burlington, MA, USA). ^15^

Real-time PCR was performed using the following method. The MSC-containing media were aspirated, rinsed two times with 1 mL of PBS, and then added with 500 μL of RNAiso/well. Afterward, 100 μL of chloroform was poured into a small tube containing scraped-off cells. Then, the tube was repeatedly shaken. Centrifugation was performed to separate the supernatant (250–300 μL) for 15 min at 12,000 rpm and 4°C. Only RNA molecules remained in the tube after the supernatant was removed; thus, 500 μL of 75% ethanol vortex was added until the pellet floated. At 12,000 rpm and 4°C, the samples were centrifuged for 5 min. After the supernatant was removed from the tube, it was dried for 5–10 min before adding 25 μL of Aqua bidest. The RNA concentration suitable for spectrophotometric measurement and purification was 1.7–2.1 μL in 1 μL of the tested sample. ^7^^,^^15^

The RNA sample was then added to 11 μL of Aqua bidest, and 9 μL of the combined component was placed into a new tube and mixed with 11 μL of RNA samples. Thereafter, the mixture was incubated in a PCR machine followed by pre-denaturation at 37°C for 15 min, denaturation at 65°C for 10 min, annealing at 65°C for 10 min, and final extension at 65°C for 2 h. All stages were repeated for 40 cycles. This identification was used a primary sequence template CD44f (5′-TCC CAG TAT GAC ACA TAT TGC-3′) and CD44r (5′-CAC CTT CTT CGA CTG TTG AC-3′) (Oligo Macrogen, Seoul, Korea). Real-time PCR identification was prepared for 20 e-DNA samples for 30 min. ^7^

In addition, characterization based on phenotypical characterization by flow cytometry was also carried out to evaluate CD44, CD90, CD73, CD105, and CD45. Identification was carried out by calculating CD44, CD90, CD73, CD105, and CD45 proteins in a solution that was injected into a flow cytometry device (FacsCalibur-BD with the Cellquest computerize program), and then the particles were randomly distributed in three-dimensional space. The sample must be in a stream of single particles so that it can be detected and regulated by the delivery system. Once hydrodynamics was centered, each particle was pushed towards one or more lights. The light glows in the form of a fluorescence emission when the particles are labeled with a fluorochrome (FITC, PE, APC, or PerCP-Cy5.5). In this study, several polyclonal antibodies were used, such as Rabbit Anti-CD44/HCAM/PGP1 Polyclonal Antibody, PE conjugated Primary Antibodies (bs-0521R-PE, Biossusa); Rabbit Anti-Thy-1/CD90/Thy1.1 Polyclonal Antibody, FITC conjugated Primary Antibodies (bs-0778R-FITC, Biossusa); APC anti-mouse cross anti-rabbit CD105 (120414/100 μg, BioLegend); PE anti-human cross anti-rabbit CD73 (BD); and PerCP-Cy5.5 anti-human cross anti-rabbit CD45 (BD).

### Animal model of ovarian failure

Female rats used in this study were fasted for five days but given unlimited access to water, creating a rat model with ovarian failure. ^21^^,^^24^ Female rats aged 12 to 14 weeks and weighing 250 to 300 g served as the study's model animals. In the experimental animal facility of the Faculty of Veterinary Medicine, Universitas Airlangga, the rats were housed in separate plastic cages with sufficient ventilation. ^21^

### Transplantation methods of MSCs

The transplanted MSCs were evaluated against negative and positive control rats, as well as female rats with ovarian failure. The T1 group included 15 infertile female rats that received 200 million stem cells transplanted via tail vein per rat from a four-day normoxic culture (21% O _2_ concentration). ^7^^,^^9^

The T2 group included 15 infertile female rats that received 200 million stem cells transplanted via tail vein per rat from a four-day low O _2_ culture (1% O _2_ concentration). ^7^^,^^9^ 0.1 mL of PBS injection was given to 15 healthy female rats in the fertile positive control group. A total of 15 infertile female rats were injected with 0.1 mL of PBS as part of the negative control group (infertile rat).

Ovarian tissue was collected from the ovaries of female rats 10 days after surgical excision (allowing for two estrous cycles). ^21^

Histopathological preparations using hematoxylin and eosin (HE) stain (B8438, Sigma Aldrich, Burlington, MA, USA) allowed researchers to detect an improvement in ovarian tissue. HSP70 expression (with monoclonal antibody HSP 70, catalog number MA3-007, Thermo Fisher Scientific, Waltham, MA USA) as anti-apoptosis was assessed by immunohistochemistry (IHC). Caspase-3 expression (with caspase-3 monoclonal antibody, E-8 7272 for IHC staining, Santa Cruz Biotechnology, LI-COR, Bioscience, Santa Cruz, CA, USA), as a pro-apoptotic factor, was inhibited. In addition, VEGF-1 expression (with a mouse monoclonal antibody, cone OTI4E3 [formerly 4E3], True MAB, OriGene, Beijing, China) served as a marker of the viability of stem cells, whereas GDF-9 (with a GDF-9 monoclonal antibody, sc-514933, Santa Cruz Biotechnology, LI-COR, Bioscience, Santa Cruz, CA, USA) served as a marker of the differentiation of stem cells into progenitor germ cells to improve ovarian failure and infertility. ^7^^,^^9^

### Histopathological observation

Histopathological observation of ovarian tissue in the presence of a Graafian follicle and regeneration of tissue were performed through light microscopy. ^25^

A 10% formalin solution was used to fix the tissues of the ovaries. Subsequently, the ovaries were dehydrated using a series of progressively increasing alcohol concentrations, cleaned with xylol, and fixed in paraffin. Routine staining was performed on thin sections and placed on slides. ^26^

A light microscope with 400× magnification was used to conduct histopathological evaluation. Each slide’s five fields of view were evaluated. Based on an existing histological description, a Graafian follicle was observed and identified, and seminiferous tubular regeneration was performed. ^21^

### Immunohistochemical observation ^27^

The expression of HSP70, caspase-3, VEGF-1, and GDF-9 was examined by IHC. Samples were prepared for histological examination by making an incision transversely from the paraffin blocks of ovarian tissue. IHC used monoclonal antibodies to analyze the expression of HSP70 (catalog number MA3-007, HSP 70 monoclonal antibody, Thermo Fisher Scientific, Waltham, MA, USA), caspase-3 (caspase-3 E-8 7272 monoclonal antibody for IHC staining, Santa Cruz Biotechnology, LI-COR, Bioscience, Santa Cruz, CA, USA), VEGF-1 (mouse monoclonal antibody, cone OTI4E3 [formerly 4E3], True MAB, OriGene, Beijing, China), and GDF-9 (sc-514933, GDF-9 monoclonal antibody, Santa Cruz Biotechnology, LI-COR, Bioscience, Santa Cruz, CA, USA). Observations were performed using a light microscope with 200× magnification. Each slide’s five fields of view were evaluated to determine the score of tissue with brownish chromogen as a result of HSP70, caspase-3, VEGF-1, and GDF9-9. The IHC scoring system ^27^ assigned an IHC score of A×B (A=width of expression percentage; B=intensities of chromogen color; Table 1).

**Table 1.** Semiquantitative immunohistochemistry (IHC) scale of the percentage of positive cells (A) and intensity of reaction color (B) with the final score representing the product of the two variables (A×B). ^27^

A	B
0 pt no cells with positive reaction	0 point (pt) no color reaction
1 pt to 10% cells with positive reaction	1 pt low intensity of color reaction
2 pts 11%–50% cells with positive reaction	2 pts moderate intensity of color reaction
3 pts 51%–80% cells with positive reaction	3 pts intense color reaction
4 pts >80% cells with positive reaction	

### Statistical analysis

The expression of HSP70, caspase-3, VEGF-1, and GDF-9 and the Graafian follicle count with a 99% confidence level (= 0.01) and 0.05 significance threshold of difference (p<0.05) were statistically analyzed using SPSS (v. 17 for Windows XP; SPSS Inc, Chicago, IL, USA). The comparative steps for hypothesis testing included normality data test, Kolmogorov–Smirnov test, homogeneity of variance test, analyses of variance, and Tukey’s significant difference post hoc test with 5% least significant difference.

## Results

Data were collected from 60 female rats. The data were divided into four groups of treatment: (1) fertile female (normal rat) as the positive control group; (2) infertile female treated with PBS as the negative control group; (3) infertile female with stem cells transplanted from the four-day normoxic culture (21% O _2_ concentration) as the T1 group (first treatment); (4) infertile female with stem cells transplanted from the four-day low-O _2_ culture (1% O _2_ concentration) as the T2 group (second treatment). The study results revealed that MSCs transplantation from the hypoxic precondition culture improved ovarian function by decreasing the damage level and increasing fertility. The expression level of HSP70, VEGF-1, and GDF-9 was upregulated, whereas the expression level of caspase-3 was decreased; the regeneration of ovarian tissue achieved a normal histopathological figure as evidenced by the development of a follicle into a Graafian follicle.

### The effects of hypoxia on MSCs

***MSCs characterization***

**Figure 1.**
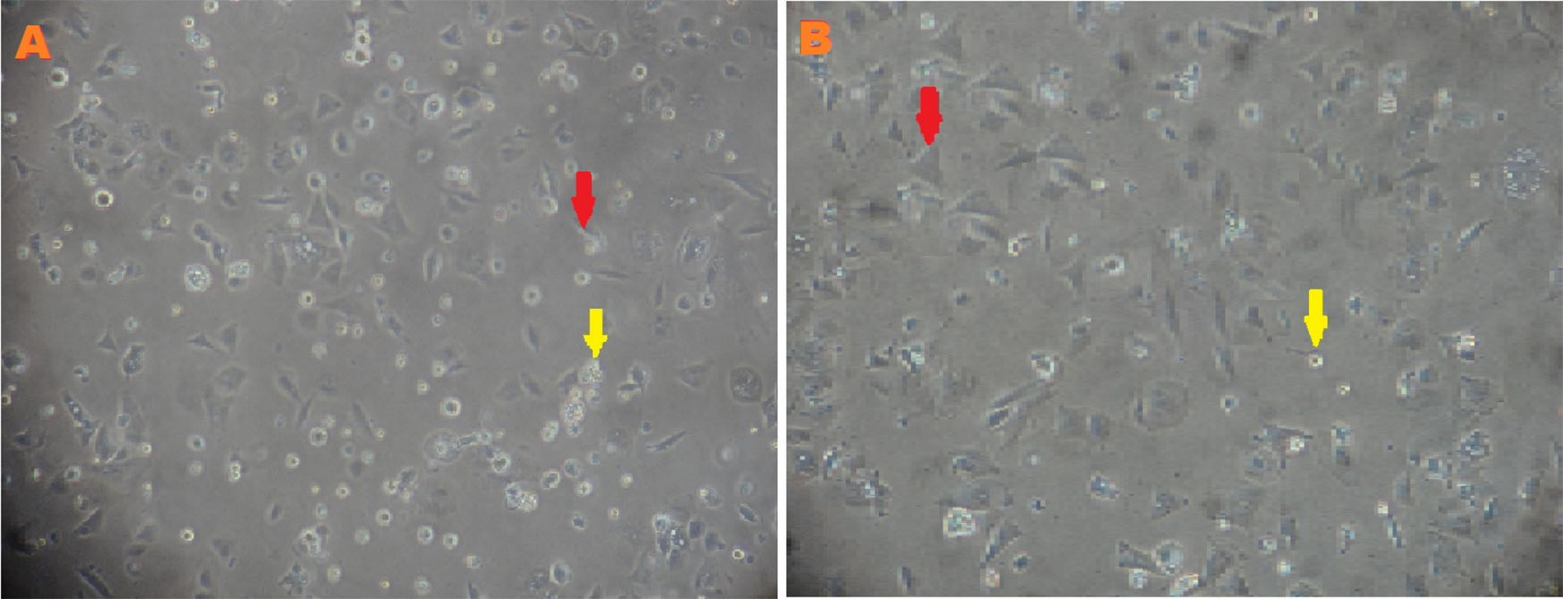
Morphology of mesenchymal stem cells (MSCs) after third passage at two days in normoxia (A) and hypoxia (B) with α MEM + 10% FBS medium cultures. MSCs (red arrow) indicated a spindle-shaped or fibroblastic morphology, meanwhile, cell death (yellow arrow) indicated an Amorphous form. Magnification at 400×.

Characteristics of MSCs between low-O _2_ and normoxic cultures were observed before being transferred into the ovaries to assess cell morphology, HSP27 and caspase-3 expression, and apoptosis. MSCs characterization was performed using an inverted microscope after the third passage of normoxic (O _2_ 21%) and low-O _2_ (O _2_ 1%) cultures on the second day. The cells exhibited a larger cell size, fewer cell deaths, and slower proliferation rate in the low-O _2_ culture, whereas the cells showed a higher rate of proliferation, more cell deaths, and smaller cell sizes in the normoxic culture ( Figure 1).

**Figure 2.**
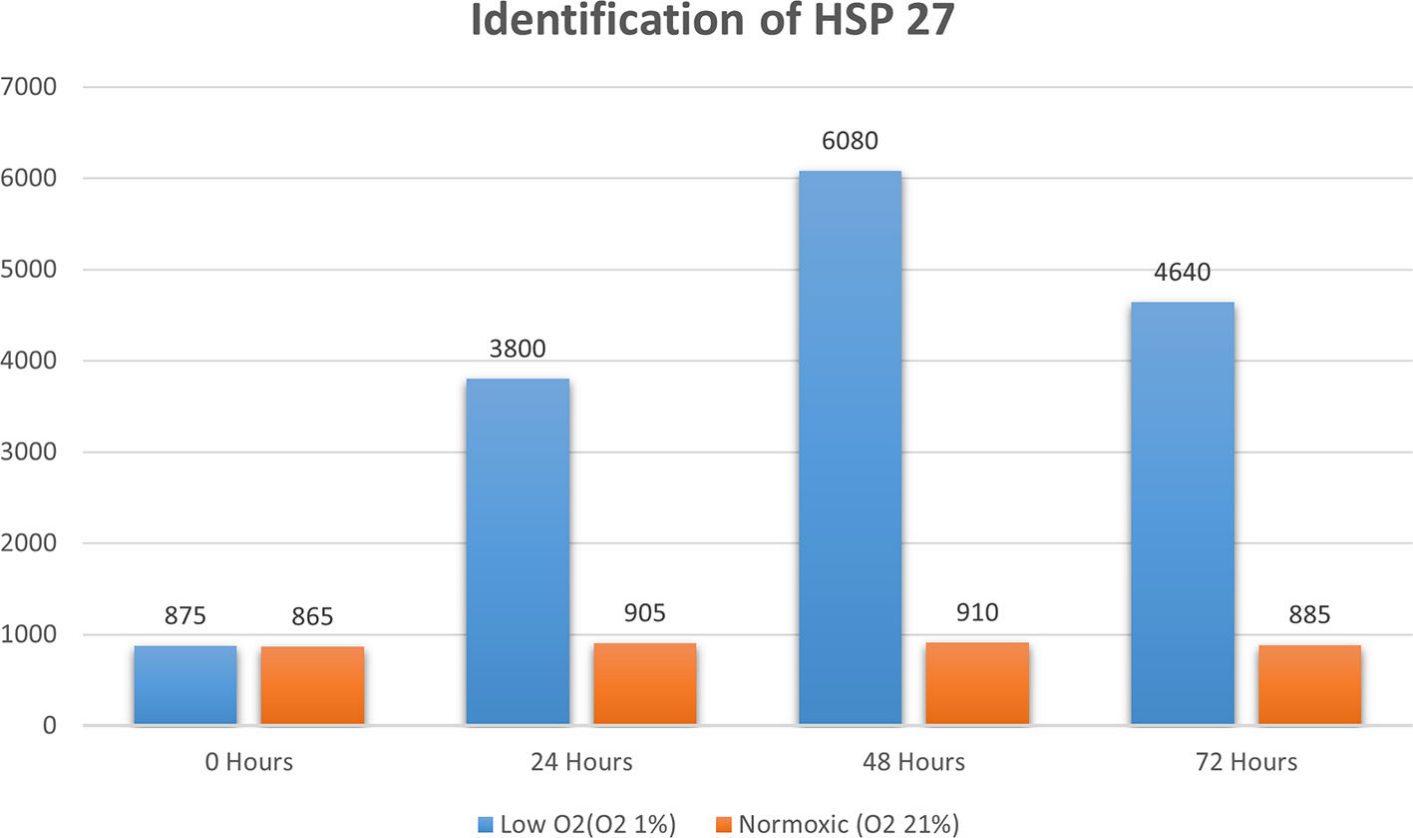
Identification of HSP 70 by using real-time PCR showed significantly increasing HSP 70 expression ( *P*<0.05) in hypoxic culture (O _2_ 1%) for 48 h compared to normoxic culture (O _2_ 21%).

**Figure 3.**
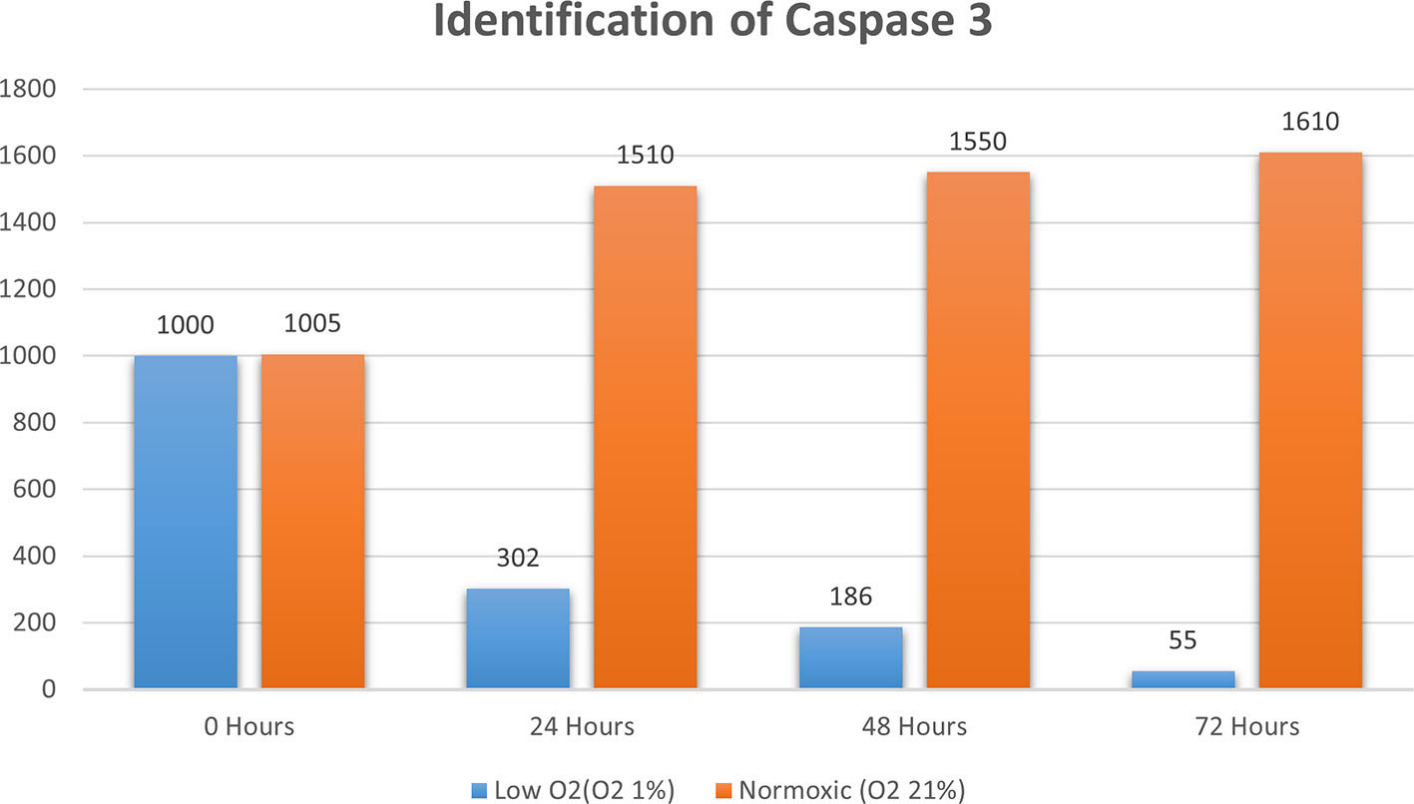
Identification of caspase 3 by using RT-PCR showed significantly decreasing caspase 3 expression ( *P*<0.05) in hypoxic culture (O _2_ 1%) compared to normoxic culture (O _2_ 21%).

Real-time PCR analysis of HSP27 and caspase-3 expression revealed considerably and significantly increased HSP27 and decreased caspase-3 under low oxygen tension (O _2_ 1%; p<0.01) compared with those under the normoxic culture as a control ( Figures 2 and 3).

**Figure 4.**
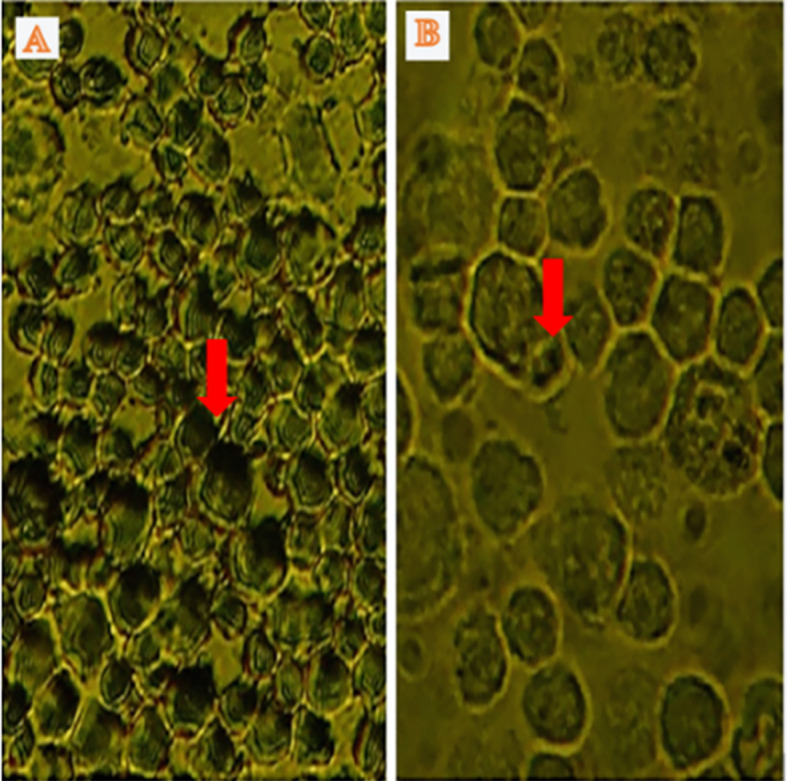
Identification of apoptosis using immunocytochemistry in normoxic and low-O _2_ conditions. (A) Positive expression of apoptosis in normoxia (red arrow) and number of apoptotic cells reaching 25%. (B). Low-O _2_ culture *in vitro* for 72 h show a small number of apoptotic cells (5%, red arrow). Magnification at 400×.

Apoptosis was detected through IHC under normoxic and low-O _2_ conditions. The positive expression of apoptosis reached 50% of cells cultured under a normoxic condition, whereas only 5% of cells cultured under low O _2_ culture *in vitro* until 72 h exhibited apoptotic cells ( Figure 4).

**Figure 5.**
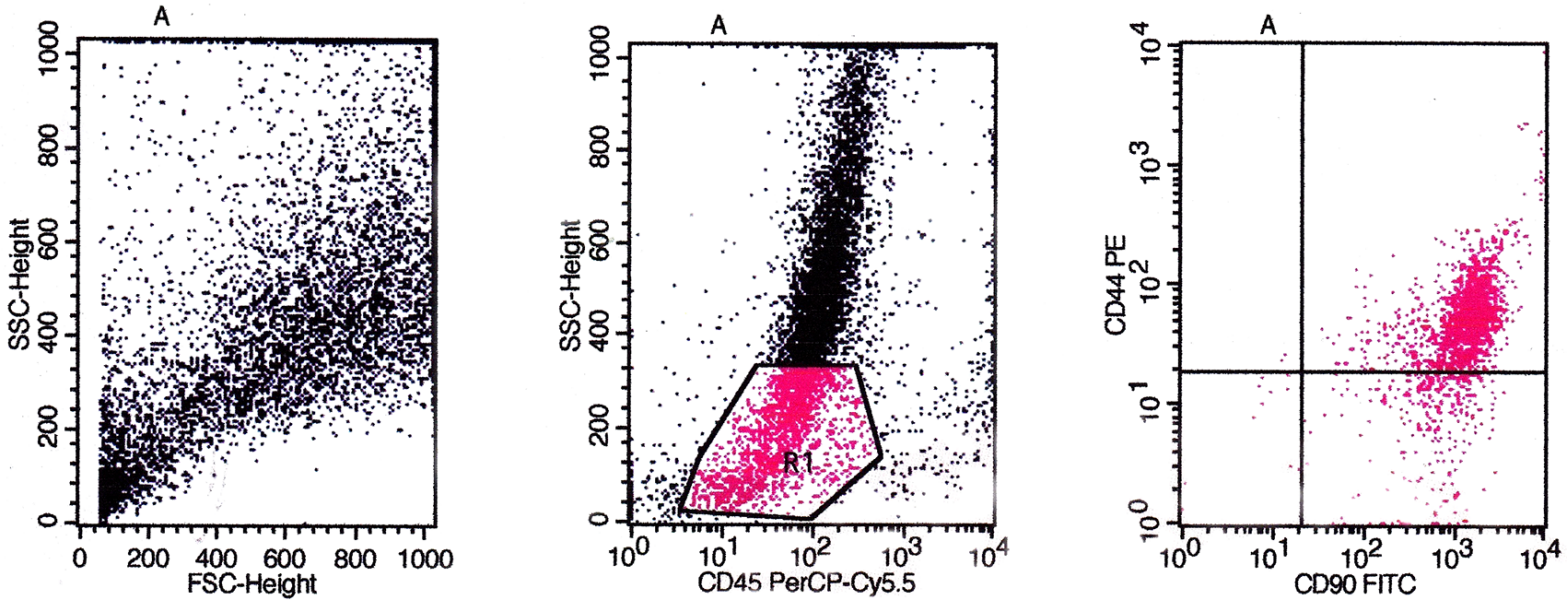
Identification of CD45/CD90/CD44 using flow cytometry before cells were cultured in low O _2_.

**Figure 6.**
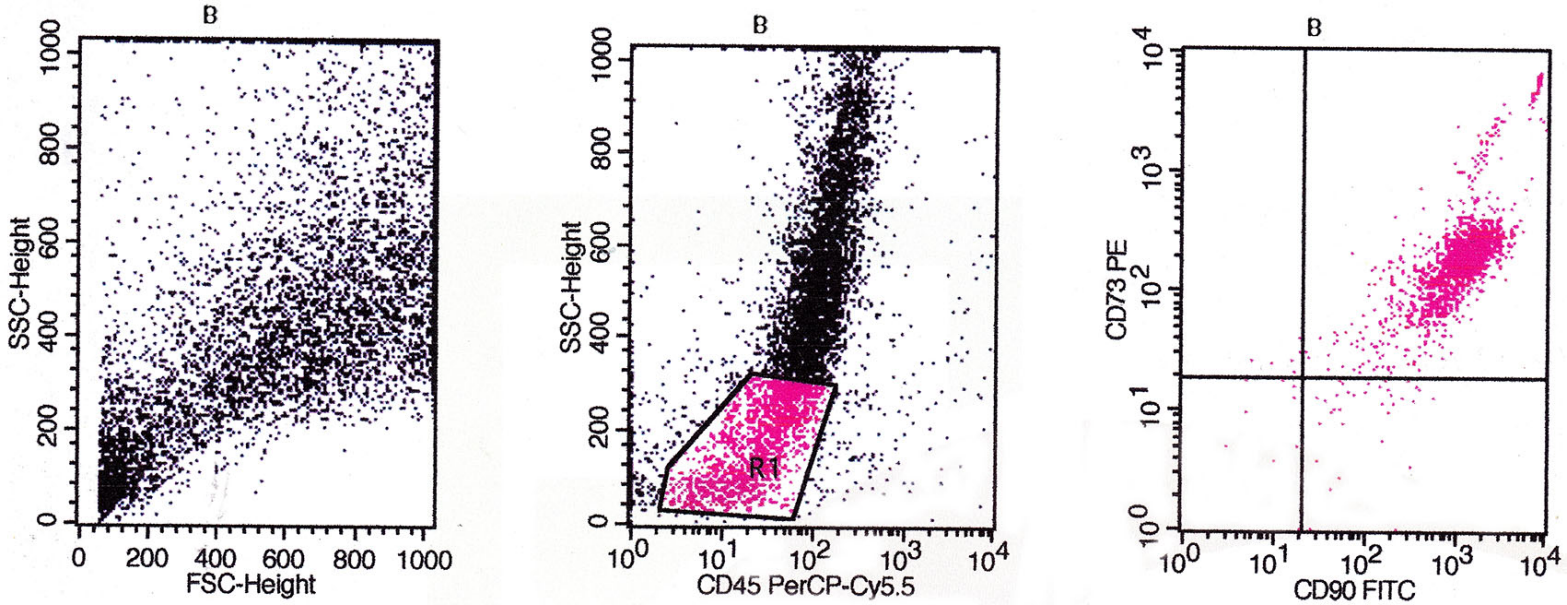
Identification of CD45/CD90/CD73 using flow cytometry before cells were cultured low O _2_.

**Figure 7.**
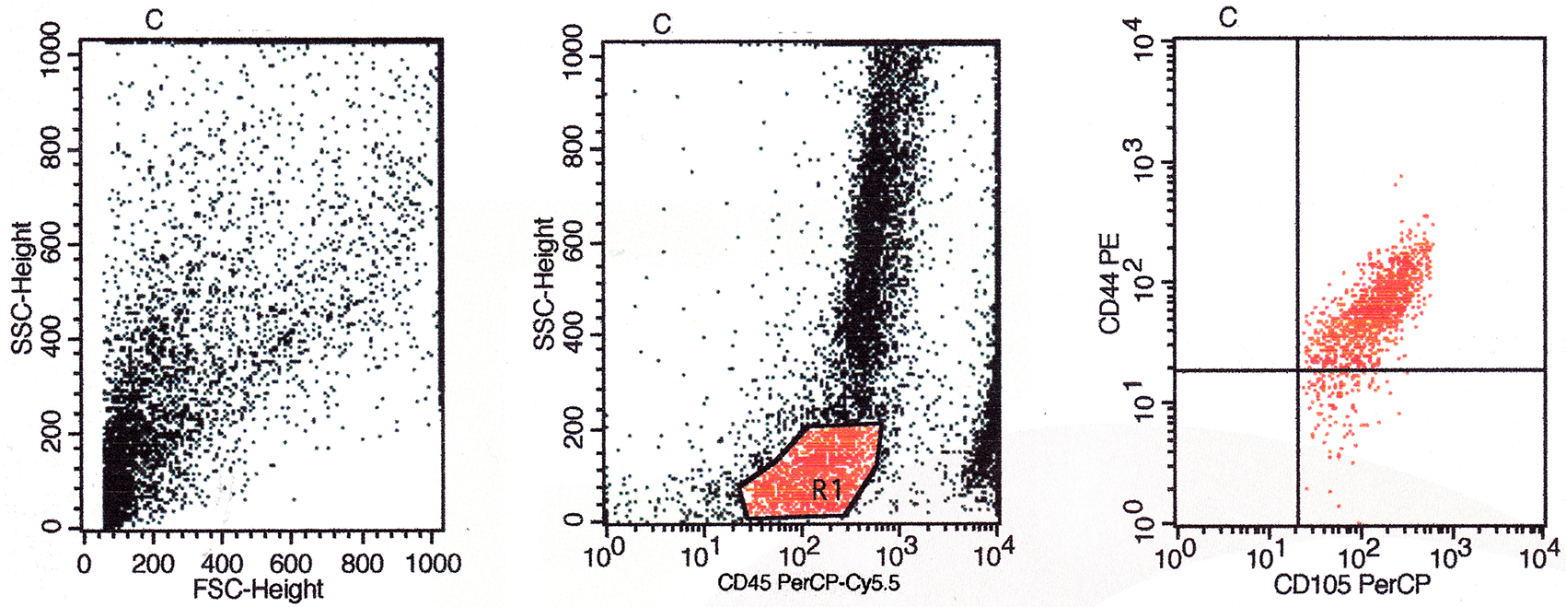
Identification of CD45/CD105/CD44 using flow cytometry before cells are cultured in low O _2_.

Based on flow cytometry evaluation, the phenotype of CD44, CD90, CD73, CD105, and CD45 were used as positive markers and negative markers for MSCs from bone marrow. The identification results obtained based on the flow cytometry method showed that the cultured cells were MSCs ( Figures 5, 6, and 7).

The evaluation results based on Figure 5 showed the expression of CD45/CD90/CD44 on BMSCs including the number and percentage. Based on quadrant statistics in the upper right (UR) area the number of CD44 and CD90 was 1852 (84.21%), while in the lower right (LR) area the number of CD90 was 335 (15.20%) with the number of cells obtained being 2200 (gated events).

The evaluation results based on Figure 6 showed the expression of CD45/CD90/CD73 on BMSCs including the number and percentage. Based on quadrant statistics in the upper right (UR) area the number of CD73 and CD90 was 1682 (97.06%), while in the lower right (LR) area the number of CD90 was 36 (2.08%), with the number of cells obtained being 1733 (gated events).

The evaluation results based on Figure 7 showed the expression of CD45/CD105/CD44 on BMSCs including the number and percentage. Based on quadrant statistics in the upper right (UR) area the number of CD44 and CD105 was 1205 (90.40%) while in the lower right (LR) area the number of CD105 was 128 (9.60%) with the number of cells obtained being 1333 (gated events).

### Effect of MSCs on rat model with ovarian failure

***HSP70 expression***

**Figure 8.**
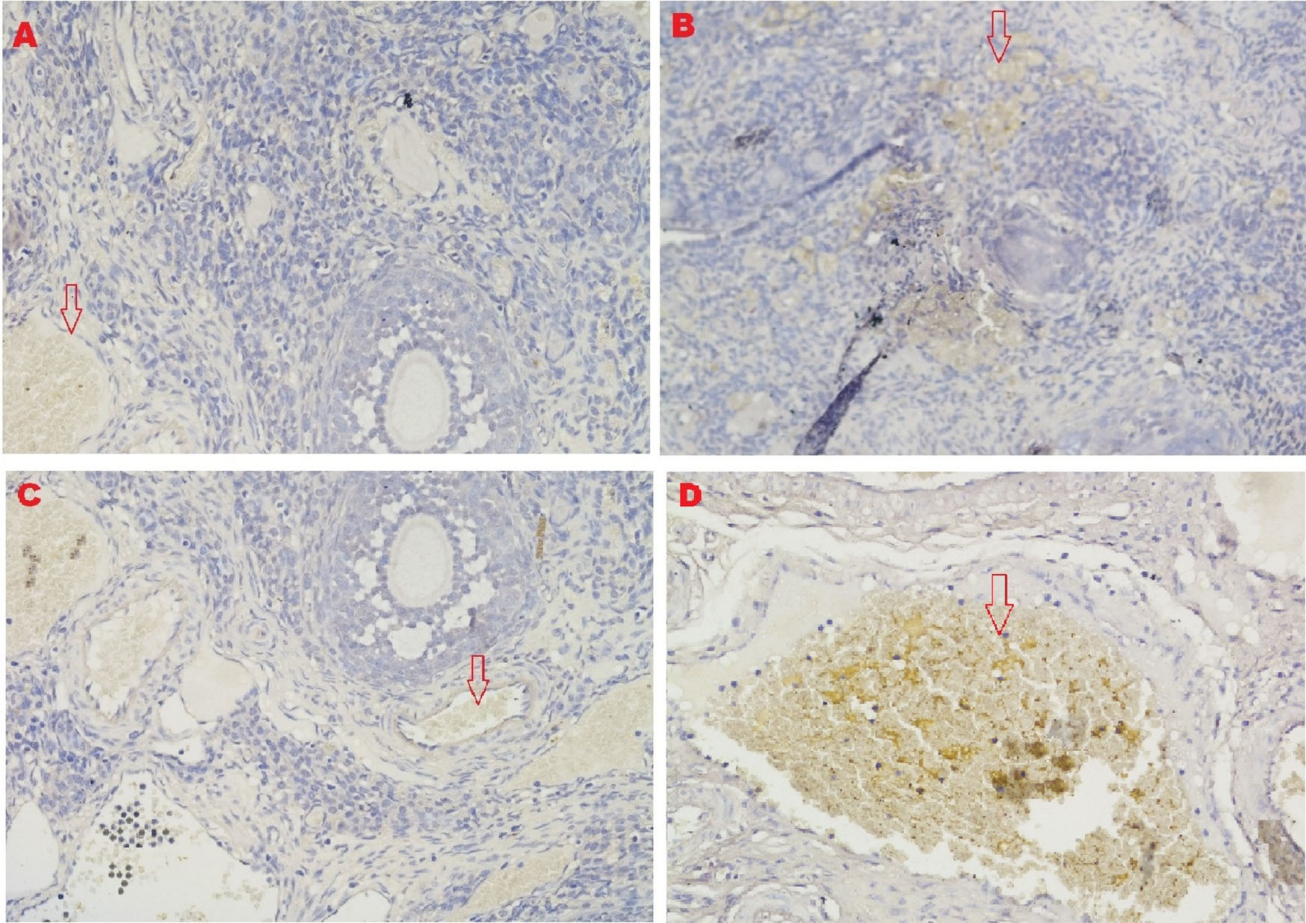
Average score of HSP70 expression is reported with brownish color intensity (red arrow): A. Positive (fertile) control group=0.5 ^a^±0.53; B. T2 group=1.7 ^a^±0.82; C. T1 group=6.2 ^b^±1.5; D. Negative (infertile) control group=9.6 ^c^±1.3. A, B, C, D: magnification at 200× with IHC.

The IHC score for HSP70 expression in ovarian tissue from four treatments is shown in Figures 2, 8 and Table 2. As shown in Table 2, the average score of HSP70 expression (brown) was 0.5 ^a^ ± 0.53 for the positive (fertile) control group, 1.7 ^a^±0.82 for the T2 group, 6.2 ^b^±1.5 for the T1 group, and 9.6 ^c^±1.3 for the negative (infertile) control group.

**Table 2.** Average score of HSP70, Caspase3, VEGF1, and GDF9 expression with immunohistochemistry and Graafian follicle count in ovarian tissue of rat.

Treatments	Mean score±SD				
	HSP70 expression	Caspase 3 expression	VEGF expression	GDF9 Expression	Graafian follicular count
Positive control group	0.5 ^a^±0.53	0.2 ^a^±0.42	10.8 ^c^±1.55	5.8 ^c^±1.47	8.9 ^c^±0.74
Negative control group	9.6 ^c^±1.3	7.3 ^c^±1.42	0.2 ^a^±0.42	0.3 ^a^±0.48	0.4 ^a^±0.52
T1 group	6.2 ^b^±1.5	4.8 ^b^±1.03	0.4 ^a^±0.52	0.5 ^a^±0.53	0.5 ^a^±0.53
T2 group	1.7 ^a^±0.82	0.6 ^a^±0.52	8.7 ^b^±0.48	4.6 ^b^±0.97	4.5 ^b^±0.71

^a–d^ Different superscripts in the same column were significantly different (p *<*0.05).

***Caspase-3 expression***

**Figure 9.**
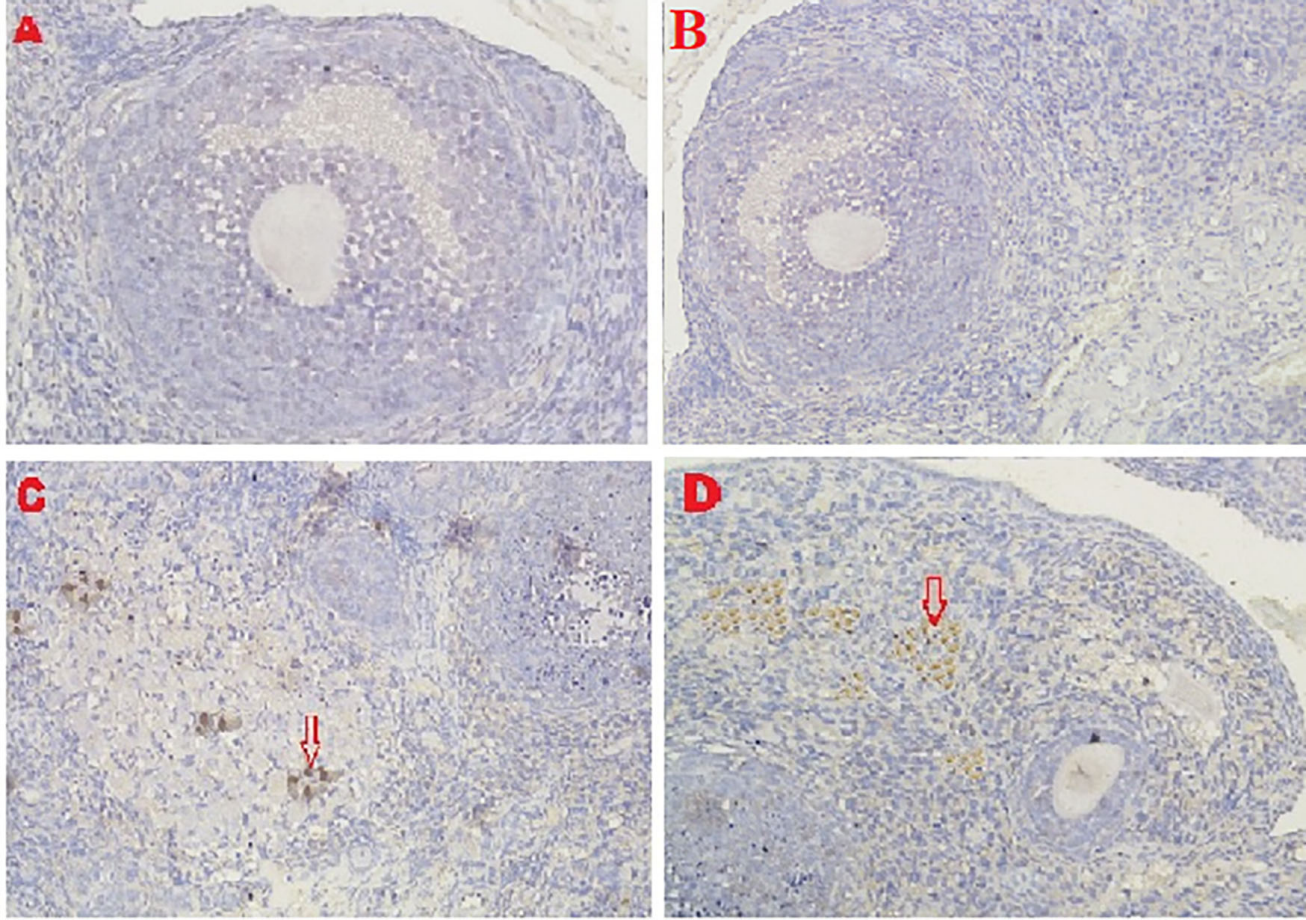
Average score of Caspase3 expression is reported with brownish color intensity (red arrow): A. Positive (fertile) control group=0.2 ^a^±0.42; B. T2 group=0.6 ^a^±0.52; C. T1 group=4.8 ^b^±1.03; D. Negative (infertile) control group=7.3 ^c^±1.42. A, B, C, D: magnification at 400× with IHC.

The IHC score for caspase-3 expression in ovarian tissue from four treatments is shown in Figures 3, 9 and Table 2. As shown in Table 2, the average score of caspase-3 expression (brown) was 0.2 ^a^±0.42 for the positive (fertile) control group, 0.6 ^a^±0.52 for the T2 group, 4.8 ^b^±1.03 for the T1 group, and 7.3 ^c^±1.42 for the negative (infertile) control group.

***VEGF-1 expression***

**Figure 10.**
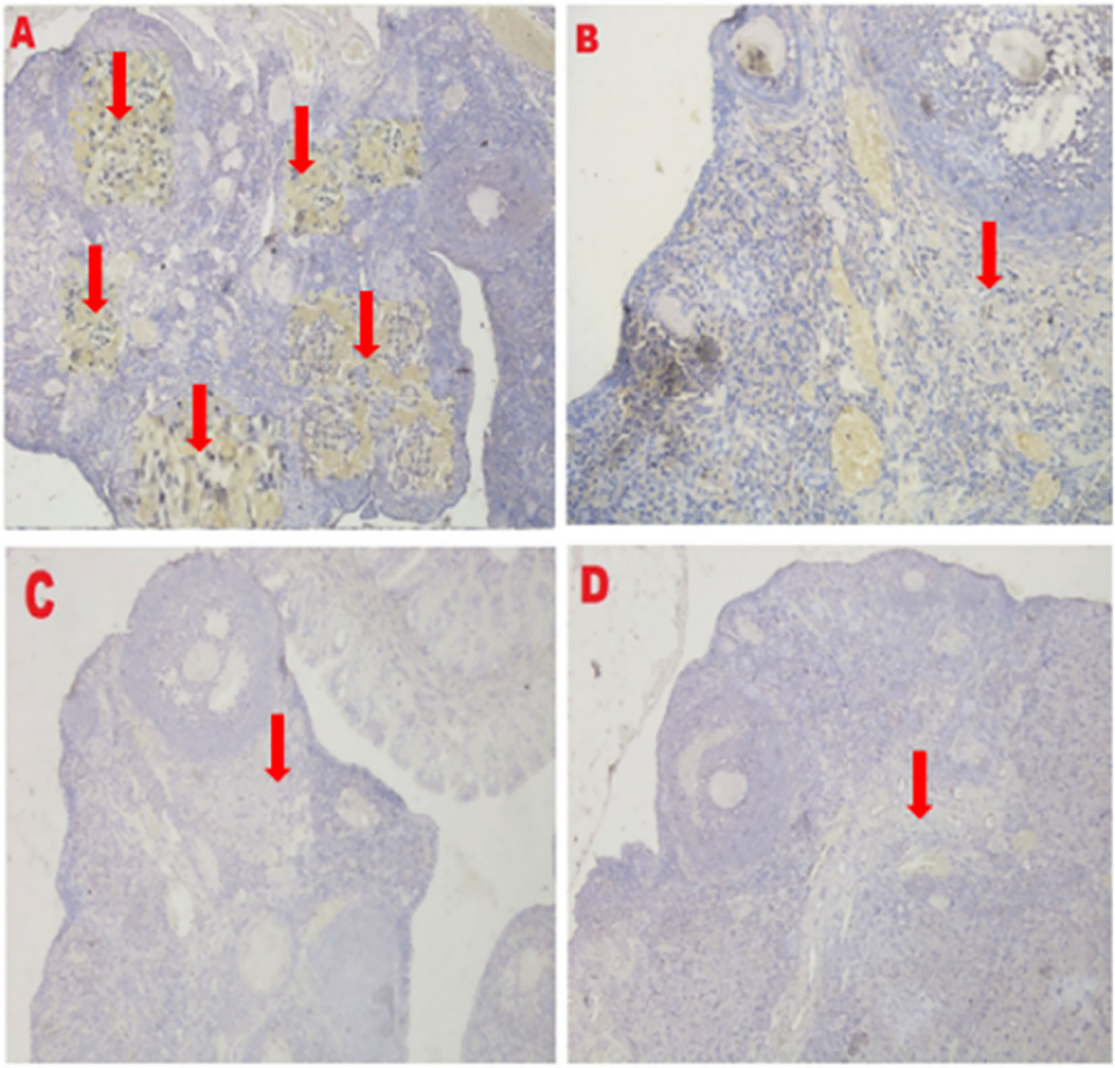
Average score of VEGF-1 expression is reported with brownish color intensity (red arrow): A. Positive (fertile) control group=10.8 ^c^±1.55; B. T2 group=8.7 ^b^±0.48; C. T1 group=0.4 ^a^±0.52; D. Negative (infertile) control group=0.2 ^a^±0.42. A, B, C, D: magnification at 400× with IHC.

The IHC score of VEGF-1 expression in ovarian tissue from four treatments is shown in Figure 10 and Table 2. As shown in Table 2, the average score of VEGF-1 expression (brown) was 10.8 ^c^±1.55 for the positive (fertile) control group, 8.7 ^b^±0.48 for the T2 group, 0.4 ^a^±0.52 for the T1 group, and 0.2 ^a^±0.42 for the negative (infertile) control group.

***GDF-9 expression***

**Figure 11.**
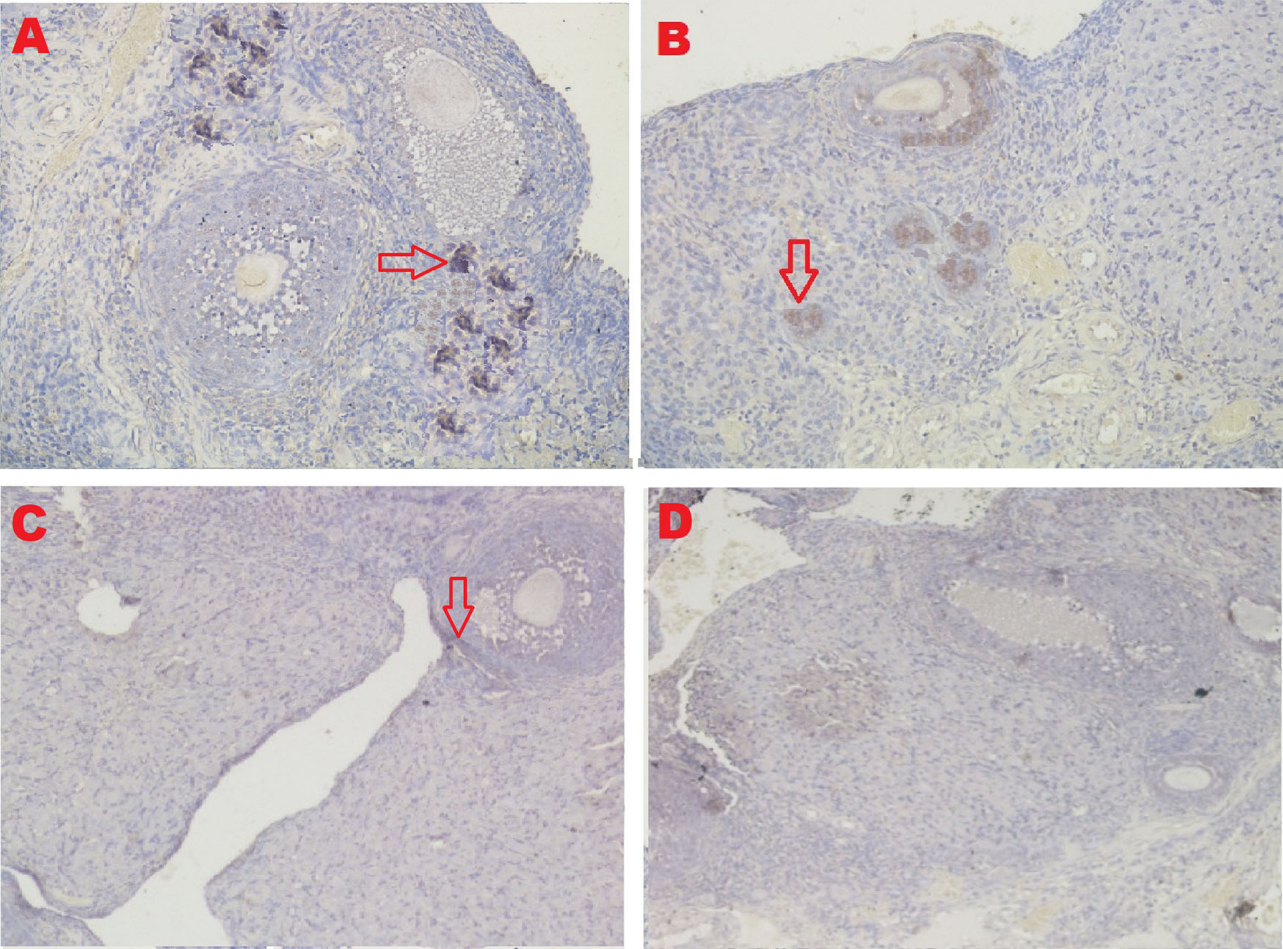
Average score of GDF-9 expression is reported with brownish color intensity (red arrow): A. Positive (fertile) control group=5.8 ^c^±1.47; B. T2 group=4.6 ^b^±0.97; C. T1 group=0.5 ^a^±0.53; D. Negative (infertile) control group=0.3 ^a^±0.48. A, B, C, D: magnification at 400× with IHC.

The IHC score of the GDF-9 expression in ovarian tissue from four treatments is shown in Figure 11 and Table 2. As shown in Table 2, the average score of the GDF-9 expression (brown) was 5.8 ^c^±1.47 for the positive control group (fertile rats), 4.6 ^b^±0.97 for the T2 group, 0.5 ^a^±0.53 for the T1 group, and 0.3 ^a^±0.48 for the negative control group (infertile rats).

***Ovarian regeneration***

**Figure 12.**
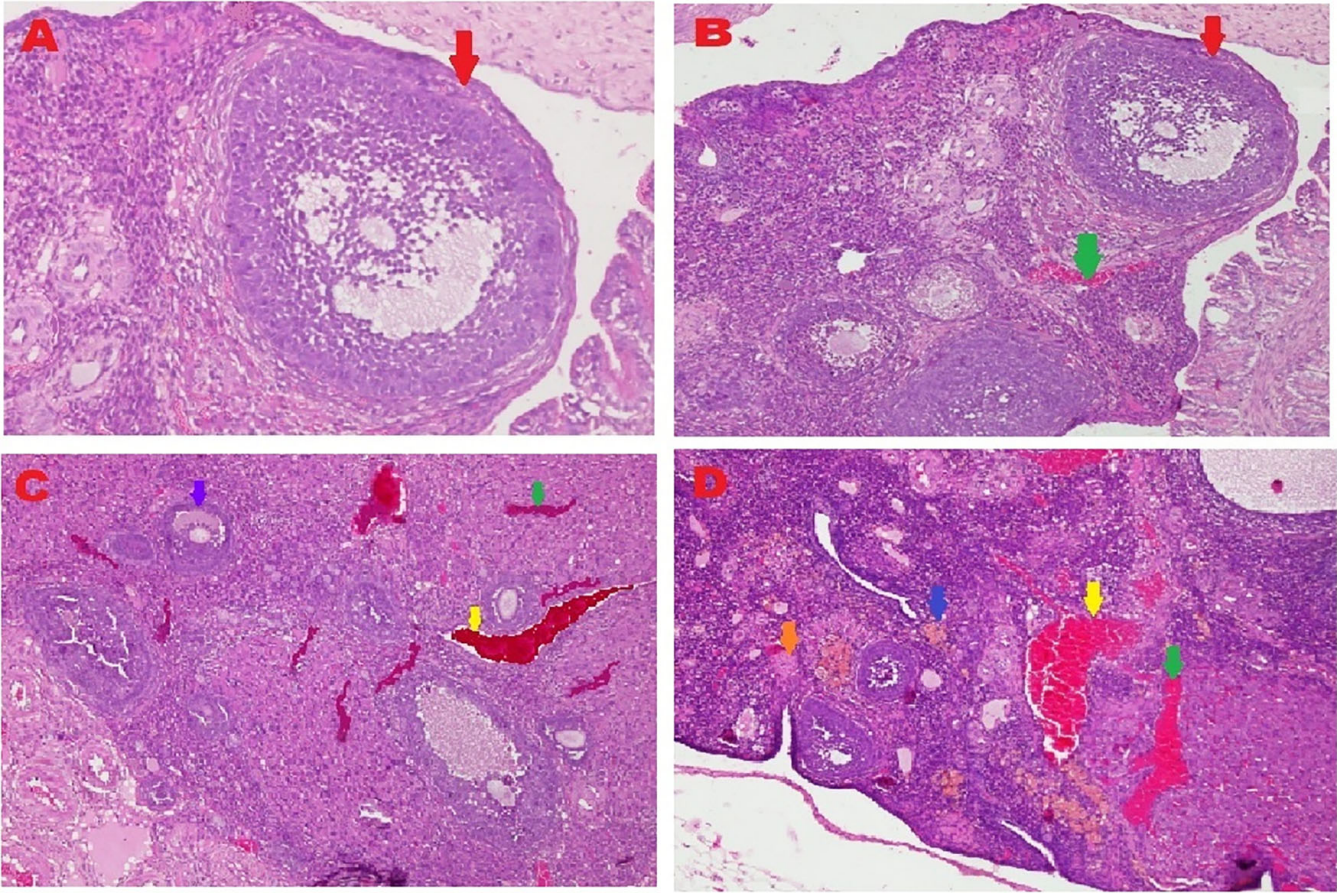
Regeneration of ovarian tissue and improvement of Graafian follicle count indicated by microscopic examination with hematoxylin and eosin (HE) staining in rat ovarian tissue in four treatments and magnification at 400×: **A.** Positive (fertile) control group showed Graafian follicle ( 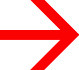), with an average count of 8.9 ^c^±0.74; **B.** T2 group, ovaries begin to regenerate and become intact despite the presence of hemorrhage ( 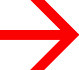) in some areas, but a Graafian follicle ( 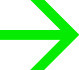) was observed with an average count of 4.5 ^b^±0.71; **C**. T1 group, ovarian congestion ( 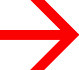), severe hemorrhage ( 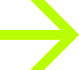), and growing follicles ( 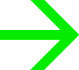) were observed, but no Graafian follicle was found (0.5 ^a^±0.53); **D**. Negative (infertile) control group, ovarian congestion ( 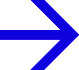), severe hemorrhage ( 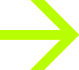), and visible hemosiderin ( 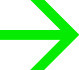) caused by blood cell lysis (brownish yellow) with fibrin deposition ( 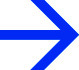) were observed, indicating that chronic congestion occurred (pink) without growing follicles or Graafian follicle (0.4 ^a^±0.52).

The microscopic examination from five different angles showed that the T2 group had a repaired tissue ovary. As shown in Figure 12, an improvement in ovarian tissue regeneration and Graafian follicle count was detected via microscopic examination with 400× magnification using HE staining of rat ovarian tissue from the four treatment groups. The positive (fertile) control group exhibited a Graafian follicle without hemorrhage, congestion, hemosiderin, or deposition of fibrin in the ovaries. The T2 group exhibited the regeneration of intact ovaries despite hemorrhage in some areas; however, a Graafian follicle had developed. The T1 group showed ovarian congestion and severe hemorrhage, although follicles were developed. Moreover, no Graafian follicle was observed. The negative (infertile) control group exhibited ovarian congestion, severe hemorrhage, and hemosiderin because of blood cell lysis (brownish yellow) with fibrin deposition, which indicated the presence of chronic congestion (pink colour); no follicles or Graafian follicle had developed.

### Improvement of the Graafian follicle count

A Graafian follicle count for ovarian tissue from each of the four treatments was conducted using HE staining ( Figure 12 and Table 2). As shown in Figure 12, the Graafian follicle count obtained by microscopic examination at 400× magnification using HE staining of rat ovarian tissue from each of the four treatments were as follows: the positive (fertile) control group displayed an average count of 8.9 ^c^±0.74; the T2 group displayed an average count of 4.5 ^b^±0.71; the T1 group showed growing follicles but no Graafian follicle (=0.5 ^a^±0.53); the negative (infertile) control group showed no growing follicles nor Graafian follicle (=0.4 ^a^±0.52).

## Discussion

Low O _2_ tension is a crucial element in the stem cell microenvironment, ^15^ which is important for stem cells self-renewal, viability, and proliferation stability. In this research, the effect of low O _2_ on cell proliferation was observed on the basis of morphological analysis of MSCs. After the third passage on the second day of culturing under low O _2_, cells exhibited a larger cell size, fewer cell deaths, and a slower proliferation rate; meanwhile, cells in the normoxic culture exhibited a smaller size, higher cell deaths, and faster proliferation rate ( Figure 1). This research was consistent with a previous study, ^28^ which reported that the slow proliferation of stem cells is necessary for the inhibition of senescence cells or rapidly aging cells. Another study has reported that rapid proliferation is needed to achieve cell confluence and prevent cell deaths. ^15^

The results of this research indicated that low O _2_ can inhibit apoptosis. Based on real-time PCR, the expression of HSP27 and caspase-3 was detected. This result indicated significantly increased HSP27 and decreased caspase-3 expressions in cells *in vitro* cultured with low oxygen tension (O _2_ 1%, p<0.05) compared with those in cells cultured under normoxic conditions, which served as the control ( Figures 2 and 3). This research was consistent with a previous study, ^15^ which reported that low O _2_ had a significant effect on apoptotic resistance, as indicated by the expression of HSP27. The study indicated that low O _2_ could reduce apoptosis by increasing HSP27 expression and decreasing caspase-3 expression. Caspase-3 is a marker of apoptosis; thus, a decrease in caspase-3 indicated that low O _2_ could reduce apoptosis. ^20^

Furthermore, IHC of cells cultured under low-O _2_ (O _2_ 1%) conditions until 72 h exhibited a small number of apoptotic cells (5%) compared with those cultured under a normoxic condition (O _2_ 21%), which showed an apoptotic cell number of 25% ( Figure 4). The study results were supported by several studies that stated that under low-O _2_ (O _2_ 1%) conditions, the stem cells decreased the level of apoptosis. ^21^^,^^22^ The incidence of apoptosis was between 2.45% and 2.55% under low O _2_ for 24, 48, and 72 h compared with a number of cells that were cultured under normoxic (O _2_ 21%) conditions, in which the apoptotic cell number reached 12.5%. ^23^^,^^24^ *In vitro* culture of stem cells required the same treatment as those maintained *in vivo* because stem cells require a hypoxic condition (a low concentration of O _2_) to maintain self-renewal, ^25^ viability, ^26^ and slow proliferation based on the location and type of the stem cells. ^27^^,^^28^

The flow cytometry method is a method for identifying specific markers on the surface of MSCs cells qualitatively and quantitatively. The markers used for this method are monoclonal antibodies PE anti-rabbit CD44 (Biossusa), FITC anti-rabbit CD90 (Biossusa), PE anti-human cross anti-rabbit CD73 (BD), APC anti-mouse cross anti-rabbit CD105 (Biolegend), and PerCP-Cy5.5 anti-human cross anti-rabbit CD45 (BD). The identification results obtained based on the flow cytometry method showed that the cultured cells were MSCs ( Figures 5, 6, and 7).

A low-O _2_ culture of stem cells maintained the proliferation rate ^25^ and viability ^26^ and achieved self-renewal because stem cells require a low-O _2_ concentration. ^29^^–^^31^ Hematopoietic stem cells require an O _2_ concentration of 0%–5%; ^24^ adipose stem cells require an O _2_ concentration of 5%; ^32^ neural stem cells require an O _2_ concentration of 1%–5%; ^33^ cord blood requires an O _2_ concentration of 3%, ^34^ and MSCs require an O _2_ concentration of 0.5%–3%. ^35^

In nearly every type of tissue that makes up the body of multicellular creatures, stem cells may be considered as an undifferentiated cell. ^36^ However, in this study, endogen stem cells cannot repair ovarian tissues under chronic conditions because of severe malnutrition. Therefore, stem cell transplantation from an *in vitro* culture that simulates endogenous stem cells under low oxygen conditions is a potential therapy option. This research investigated an *in vitro* culture of stem cells under 1% oxygen. ^37^

Stem cells have two unique characteristics: first, stem cells can renew or regenerate themselves by replicating identically through cell division. Secondly, stem cells can differentiate from other cells. Stem cells can develop into various types of mature cells, such as nerve, heart muscle, skeletal muscle, and pancreatic cells; ^38^ female gametes; ^39^ and male gametes. ^17^ This research focuses on female gamete cells. Low O _2_ is important for stem cells to maintain their differentiation ability, and plasticity. ^40^^,^^41^ Therefore, a low-O _2_ culture can promote the survival, strong attachment, and integration of stem cells into the microenvironment of original cells to achieve successful therapy.

This study reported that MSCs culture can improve the fertility of female rats with malnutrition-induced. The success of the therapy was determined on the basis of six parameters: (1) increased expression of HSP70 as a chaperone molecule for the repair of cell protein and inhibition of apoptosis, (2) decreased expression of caspase-3 for the apoptosis indicator, (3) increased expression of VEGF-1 as a marker of viability, (4) increased expression of GDF-9 as a marker of differentiation, (5) regeneration of ovarian tissue as evidenced by the intact structure of ovarian tissue and the development of follicles, and (6) improvement of the Graafian follicle count. In the previous study revealed the transplantation of MSCs from various sources and the comparable outcomes that were attained by transplanting conditioned media derived from MSCs culture. This implies that MSCs support the paracrine system and may be helpful in ovarian function restoration. In chemoablated ovaries, Very small embryonic-like stem cells (VSELs) proliferate and react favorably to PMSG therapy. When MSCs or their secretome are transplanted, these ovary surface epithelial stem cells may be able to restore non-functioning ovaries by differentiating into oocyte-like structures *in vitro*. A significant rise in primordial follicle counts upon transplanting MSCs or their secretome into the ovaries of chemoablated mice is likely to facilitate neo-oogenesis and follicle assembly from VSELs that withstand chemotherapy in the adult ovary. ^42^

Based on the result of this study, the first parameter for successful therapy was the IHC average score of the HSP70 expression in ovarian tissue from the four treatment groups ( Figure 8 and Table 2). HSP70 expression increased under T _2_ treatment (O _2_ 1%). Although this increase was inconsistent with that of the positive control group (fertile rats), this increase improved ovarian failure caused by malnutrition, as evidenced by the increase of follicle growth and Graafian follicle count. The chaperone protein HSP70 must be produced at optimal levels to heal injured cells (not exceeding the levels necessary for cell repair).

HSP70 is a protein that is strongly expressed when exposed to oxidative stressors such reactive oxygen species. This study found that malnutrition caused oxidative stress. Overexpressed HSP70 could be found in damaged cells. ^43^ The expression of HSP70 as a chaperone molecule promotes the activity of endogenous stem cells to improve the development of follicles and Graafian follicle. Both follicle types contain the ovum and estrogen hormone which are important for female reproduction. Previous studies on male rats have shown that male gametes, also known as spermatogonia stem cells, which are found in mouse testicles, can develop into progenitor cells after transplantation. ^17^ In this study, the expression of HSP70 in T2 (O _2_ 1%) improved the development of follicles and Graafian follicle of the ovaries, which was significantly different from the result of the negative control (infertile rats), but these advancements were still not on par with the positive control (fertile rats).

The second parameter for successful therapy was based on the average IHC score of caspase-3 expression ( Figure 9 and Table 2). The caspase-3 expression decreased under T2 treatment (O _2_ 1%). The decrease was consistent with that of the positive (fertile) control group, which led to an improvement in ovarian damage caused by malnutrition, as evidenced by an enhancement in follicular growth and Graafian follicle count. Oxidative stress caused by malnutrition triggers apoptosis via an inherent route in the mitochondrial component of cells. The intrinsic route requires mitochondrial activity because of the activation and release of the caspase protein into the cytosol caused by oxidative stress. A protease, namely caspase, can disassemble protein chains. In this study, malnutrition-induced oxidative stress could be due to cytochrome binding to apoptotic protease-activating factor-1 (APAF-1) released from the mitochondria; then procaspase-9 can activate caspase-9. ^44^ Caspase-9, which serves as an apoptosis initiator, is dimerized, which triggers feedback by inhibiting BCL-2 release and then binds to procaspase-3 to activate caspase-3. Then, as an executor, caspase-3 aids in the activation of cytoplasmic and endonuclease, which may fragment nuclear DNA and break down cytosol proteins in cells. The production of apoptotic bodies, which contain intracellular organelles, express phosphatidylserine, and cause phagocytosis, is the ultimate stage of fragmentation, thereby causing cell failure as a constituent of the tissue. ^45^

The third parameter for successful therapy is based on the viability of transplanted stem cells, which is assessed on the basis of VEGF-1 expression ( Figure 10 and Table 2). VEGF-1 expression increased under T _2_ treatment (O _2_ 1%). Although this increase was not consistent with the positive control group (fertile rat), it could improve ovarian damage caused by malnutrition, as evidenced by the increase in follicular growth and Graafian follicle count. VEGF-1 as a marker of viability of transplanted stem cells is expressed in ovarian tissue. ^21^ Prior research has also revealed that MSCs cultivated in hypoxia exhibited downregulation of high-mobility group box 1 (HMGB1), the apoptotic genes BCL-2 and Caspase-3, and activation of VEGF expression. Lastly, the pro-inflammatory cytokine IL-8 was downregulated in both hypoxic cultures, but only in short-term hypoxia did the levels of the anti-inflammatory cytokines IL-1ra and granulocyte-stimulating factor (GM-CSF) elevate. ^46^

The fourth parameter for successful therapy is based on the expression of GDF-9 ( Figure 11 and Table 2). GDF-9 expression increased under T _2_ treatment (O _2_ 1%). Although this increase was not consistent with the positive control group (fertile rats), it could improve ovarian damage caused by malnutrition. The growth of ovarian cortical cells is induced by the progenitor cell marker GDF-9, which is produced from germline stem cells. ^21^^,^^47^ Stem cells rapidly develop into cells that are required to respond to damage and strengthen the immune response. ^48^ The oocyte produces GDF-9, a growth factor that is a member of the TGF-ß family. GDF-9 is essential for folliculogenesis and fertility. ^49^ In addition, progenitor germ cells can address issues with folliculogenesis caused by malnutrition by repairing damaged follicles and can restore molecular communication in ovarian follicles by increasing the synthesis of SCF and GDF-9. ^21^ Oogenesis can activate homing directly through the activation of cells that have been repressed and indirectly through the stimulation of the microenvironment (niche) of injured cells. ^50^

The fifth and sixth parameters for determining the success of therapy are based on the regeneration of ovarian tissue and improvement of the Graafian follicle count ( Figure 12 and Table 2). In the positive (fertile) control group, the Graafian follicle developed. In the T2 group (O _2_ 1%), the ovaries began to regenerate and appeared intact, and a Graafian follicle was observed despite hemorrhage in some areas. In the T1 group (O _2_ 1%), ovarian congestion and severe hemorrhage were observed, and no Graafian follicle was found despite the presence of follicles. In the negative (infertile) control group, ovarian congestion and severe hemorrhage were observed, and blood cell lysis produced hemosiderin, which is brownish-yellow and deposits fibrin, indicating the occurrence of chronic congestion (pink colour), with no development of follicles or the Graafian follicle.

Ovarian tissue regeneration showed an intact ovarian tissue structure with the development of follicles and a Graafian follicle. This result indicated the effectiveness of the therapy using stem cells cultured under low O _2_ tension (1%). In this study, the regeneration of the ovarium was observed microscopically with HE staining. ^51^ Microscopic observation showed stem cell therapy cultured under low-O _2_ tension (1%, T2) achieved ovarian tissue repair. Improvements were found on the basis of the formation of follicles and a Graafian follicle and the regeneration of ovarian tissue. The positive control group (fertile rat), which did not experience ovarian failure and maintained a normal condition with developing follicles and a Graafian follicle, was contrasted with these advances ( Figure 12). The degenerative ovarian tissue of the negative (infertile) control group of rats was compared with the aberrant characteristics of the damaged ovarian tissue. The latter demonstrated chronic congestion with ovarian congestion, severe hemorrhage, and hemosiderosis (yellow brown) caused by hemolysis of red blood cells with fibrin deposition ( Figure 12).

## Conclusions

Ovarian treatment with MSCs cultured under low-O _2_ tension could improve ovarian failure caused by malnutrition in female rats based on increased HSP70 expression and decreased caspase-3 expression as apoptotic inhibitors, increased VEGF1 and GDF-9 expression as markers of viability and differentiation, regeneration of ovarian tissue, and improved count of Graafian follicle. In our perspective, further study should be carried out *in vivo* through observations on fertility tests, oestrus evaluation, or related hormone expression levels to enhance the evidence of the efficacy of ovarian treatment with MSCs cultured under low-O _2_ tension.

## Data Availability

Figshare: Graafian follicle count data sets, as well as HSP70, Caspase-3, VEGF-1, and GDF-9 results. https://doi.org/10.6084/m9.figshare.20440575. ^52^ This project contains the following underlying data:
- Data availability.xlsx (Graafian follicle number data and Caspase-3, GDF-9, HSP70 and VEGF scores) Figshare: ARRIVE Checklist MSCs therapy. https://doi.org/10.6084/m9.figshare.20440509. ^53^ Data are available under the terms of the Creative Commons Attribution 4.0 International license (CC-BY 4.0).
