# Supplementary material for: Efficacy of mesenchymal stem cells cultured low oxygen tension ameliorates apoptotic inhibitors, viability, and differentiation of ovarian tissue: A study in a rat model with ovarian failure
Source: F1000Res. 2024 Apr 16;12:24. Originally published 2023 Jan 9. [Version 3] doi: 10.12688/f1000research.124919.3 (PMC11031646; doi:10.12688/f1000research.124919.3)
Supplement: Peer review history

# Reviewer report: Reviewer response for version 3

**DOI:** 10.5256/f1000research.164389.r266765

**Published:** 2024-04-19

**Authors:** Raheleh Farahzadi

**Affiliations:** 1 Tabriz University of Medical Sciences, Tabriz, Iran

Dear Editor(s)

 All my comments have been implemented in the revised version of the manuscript.

 Kind regards

Is the work clearly and accurately presented and does it cite the current literature?

Yes

If applicable, is the statistical analysis and its interpretation appropriate?

I cannot comment. A qualified statistician is required.

Are all the source data underlying the results available to ensure full reproducibility?

No source data required

Is the study design appropriate and is the work technically sound?

Yes

Are the conclusions drawn adequately supported by the results?

Yes

Are sufficient details of methods and analysis provided to allow replication by others?

Partly

Reviewer Expertise:

Stem cell biology, and regenerative medicine

I confirm that I have read this submission and believe that I have an appropriate level of expertise to confirm that it is of an acceptable scientific standard.

# Reviewer report: Reviewer response for version 2

**DOI:** 10.5256/f1000research.159276.r238998

**Published:** 2024-02-28

**Authors:** Raheleh Farahzadi

**Affiliations:** 1 Tabriz University of Medical Sciences, Tabriz, Iran

The results presented in the manuscript entitled *“Efficacy of mesenchymal stem cells cultured low*

*oxygen tension ameliorates apoptotic inhibitors, viability, and differentiation of ovarian tissue: A study in a rat model with ovarian failure”* are in a logical sequence to that contain data to inform the readers. The manuscript is interesting for indexing **after revision.

1. At first, some grammatical points can be seen in the text of the manuscript.
2. The *“Abstract section” *should be concise.
3. The *“Introduction section” *could be extended and explained with more details.
4. The novelty and hypothesis of the research must be involved at the end of the *“Introduction section”.*
5. The figure legends must be reported with more details.
6. The manuscript is not well referenced. All parts of the *“Methods section”* should be explained in more detail. For example, the results related to the characterization of mesenchymal stem cells (multilineage differentiation and phenotypical characterization by flow cytometry) should be involved in the revised version of the manuscript.
7. Other mentioned methods should be referenced by the appropriate references.
8. Since the discussion section is one of the most important parts of the paper, this section must be improved with more attention and explanation. In the discussion section, results must be compared with other results from previous studies.

Is the work clearly and accurately presented and does it cite the current literature?

Yes

If applicable, is the statistical analysis and its interpretation appropriate?

I cannot comment. A qualified statistician is required.

Are all the source data underlying the results available to ensure full reproducibility?

No source data required

Is the study design appropriate and is the work technically sound?

Yes

Are the conclusions drawn adequately supported by the results?

Yes

Are sufficient details of methods and analysis provided to allow replication by others?

Partly

Reviewer Expertise:

Stem cell biology, and regenerative medicine

I confirm that I have read this submission and believe that I have an appropriate level of expertise to confirm that it is of an acceptable scientific standard, however I have significant reservations, as outlined above.

# Reviewer report: Reviewer response for version 2

**DOI:** 10.5256/f1000research.159276.r227855

**Published:** 2023-12-16

**Authors:** Deepa Bhartiya

**Affiliations:** 1 Epigeneres Biotech Pvt Ltd, Mumbai, India

The authors have made changes as suggested.

 It may now be accepted

Is the work clearly and accurately presented and does it cite the current literature?

Partly

If applicable, is the statistical analysis and its interpretation appropriate?

Yes

Are all the source data underlying the results available to ensure full reproducibility?

Yes

Is the study design appropriate and is the work technically sound?

Partly

Are the conclusions drawn adequately supported by the results?

Partly

Are sufficient details of methods and analysis provided to allow replication by others?

Yes

Reviewer Expertise:

I have worked on ovarian stem cells, MSCs , oogenesis from stem cells in vivo

I confirm that I have read this submission and believe that I have an appropriate level of expertise to confirm that it is of an acceptable scientific standard.

# Reviewer report: Reviewer response for version 1

**DOI:** 10.5256/f1000research.137162.r205142

**Published:** 2023-09-26

**Authors:** Zezheng Pan

**Affiliations:** 1 Nanchang University, Nanchang, China

I regret that this article cannot be aindexed due to the problems stated below.

 This study demonstrated the protective effect of hypoxic conditions on the cell viability and differentiation capacity of bone marrow mesenchymal stem cells and confirmed the promising therapeutic potential of MSCs cultured in low O2 in rat model of malnutrition-induced ovarian failure. These works are a little innovative in providing the possibility of overcoming the original limitations of stem cell transplantation therapy for ovarian failure. This report falls short in terms of content presentation, experimental results demonstrability, legend labelling, and control group settings. Specifically, the following points:

1. Misstatements: In abstract, "Apoptotic inhibitor" should be replaced with "Apoptosis indicator". In introduction, there's a misrepresentation in “Low O2 tension caused by in vitro culture reduces apoptosis, viability, and differentiation of stem cells".
2. The setting of T1 and T2 experimental groups in abstract does not match the later text.
3. Caspase 3 promotes apoptosis and is not an inhibitor of apoptosis.
4. There is no experimental content related to "fertility score" mentioned in the results.
5. The experimental results in Figure 1 cannot well prove the relationship between cell size, death and proliferation rate and the oxygen levels in the culture environment, you can add EDU, TUNEL and other related experiments and quantify the results after statistical analysis to increase the persuasive power of the experiment.
6. Figure 2 and Figure 3 are missing units in the vertical coordinates (e.g., initial copy number of samples), and the normoxic environment group should be set as a control.
7. Figure 4 does not contain the arrows depicted in the legend, the normoxic incubation group does not specify the incubation time, and the experimental data lacks statistical analysis.
8. The presentation of the result graphs needs to be adjusted by stitching related pictures together (e.g., Figure 3 with Figure 6)
9. Fertility test, oestrus evaluation or related hormone expression level measurement could be added to enhance the persuasiveness of the conclusion.
10. The title is not clearly expressed and does not accurately reflect the research content of the article; it is recommended that the title be replaced.

Is the work clearly and accurately presented and does it cite the current literature?

Yes

If applicable, is the statistical analysis and its interpretation appropriate?

Partly

Are all the source data underlying the results available to ensure full reproducibility?

Yes

Is the study design appropriate and is the work technically sound?

Partly

Are the conclusions drawn adequately supported by the results?

Partly

Are sufficient details of methods and analysis provided to allow replication by others?

Yes

Reviewer Expertise:

Female reproduction

I confirm that I have read this submission and believe that I have an appropriate level of expertise to state that I do not consider it to be of an acceptable scientific standard, for reasons outlined above.

# Reviewer report: Reviewer response for version 1

**DOI:** 10.5256/f1000research.137162.r192203

**Published:** 2023-09-26

**Authors:** Deepa Bhartiya

**Affiliations:** 1 Epigeneres Biotech Pvt Ltd, Mumbai, India

Authors have studied the effects of transplanting MSCs (cultured in normal or hypoxic conditions) in rat ovaries with POF induced by fasting for 4 days. The article is not written nicely. It is not clear whether the cells were transplanted directly in the ovaries or via tail vein.

 The concept of culturing MSCs in hypoxic conditions is not new. It is well known in literature and should be mentioned in the Introduction.

 Results are broadly described on the effects of hypoxia on MSCs and effect of MSCs on POF ovaries. These should be written under distinct subheadings to avoid confusion. No scale bars on the images. Legends mention A, B etc and the figures are not marked. CD44 markers are mentioned in Methods – but no results are described. Results in Fig 4 are not clear. 

 If the authors think hypoxia improves the quality of transplanted MSCs, it would be best to study conditioned medium from the two conditions (normal and hypoxic conditions) and show the difference in cytokines/ growth factors released. Transplanted MSCs provide paracrine support. It is the resident stem cells/ primordial follicle reserve that will grow when MSCs are transplanted. Hypoxic conditions led to increased HSP27, decreased Caspase expression and reduced apoptosis in the MSCs. Fig 1 – it is mentioned that yellow arrows mark cell death, but that may not be true. These could be a sub-population of stem cells that get enriched in hypoxic conditions. MUSE cells!

 There is increased vasculature (Fig 9) in T1 and Negative (infertile) group. VEGF expression results in Fig 7 are not convincing. Will not Fig 7 C & D should show more VEGF? Which cells in Fig 7 A & B express VEGF? It is expressed by endothelial cells – not sure if the cells shown positive in Fig 7 A & B are endothelial cells. Using VEGF as a marker for viability may not be appropriate. It marks angiogenesis – not viability. 

 Same is the case with GDF9 expression in Fig 8. It is a stem cells and oocyte marker. Not sure what cells are shown positive in the Fig. 

 Discussion is nothing but description of results again. All the results should be shifted to the results section and discussion should be overall. It needs to discussed how MSCs help to regenerate. Fasting resulted in the loss of follicles. How they get restored on transplantation of MSCs cultured in hypoxic conditions. Authors may refer to Bhartiya *et al., *(2022) ^1^

Is the work clearly and accurately presented and does it cite the current literature?

Partly

If applicable, is the statistical analysis and its interpretation appropriate?

Yes

Are all the source data underlying the results available to ensure full reproducibility?

Yes

Is the study design appropriate and is the work technically sound?

Partly

Are the conclusions drawn adequately supported by the results?

Partly

Are sufficient details of methods and analysis provided to allow replication by others?

Yes

Reviewer Expertise:

I have worked on ovarian stem cells, MSCs , oogenesis from stem cells in vivo

I confirm that I have read this submission and believe that I have an appropriate level of expertise to confirm that it is of an acceptable scientific standard, however I have significant reservations, as outlined above.

## References

[1] : Very small embryonic-like stem cells (VSELs) regenerate whereas mesenchymal stromal cells (MSCs) rejuvenate diseased reproductive tissues. *Stem Cell Rev Rep* .2022;18(5) : 10.1007/s12015-021-10243-6 1718-1727 10.1007/s12015-021-10243-6 34410593
